# Montenegro skin test: Distracted by the promise of modernity?

**DOI:** 10.1590/0037-8682-0492-2022

**Published:** 2023-01-23

**Authors:** Mady Malheiros Barbeitas

**Affiliations:** 1 Casa de Oswaldo Cruz, Fiocruz, Rio de Janeiro, RJ, Brasil.; 2 Institut National de Santé et Recherche Médicale (Inserm), Centre de recherche médecine, sciences, santé, santé mentale, société (Cermes 3), Villejuif, France.

In 2013 the Brazilian National Health Surveillance Agency (ANVISA) determined that the leishmanin and tuberculin skin tests (LST and TST, respectively) were vaccine-like products, which redefined the manufacturing requirements for these reagents and resulted in what was then considered a temporary suspension of production of the LST (also known as the Montenegro test)^1^. At that time, the Immunology Production and Research Center (CPPI) was the only certified manufacturer of this test in Brazil and worldwide. Other institutions in Iran and Italy have stopped production due to a lack of interest and/or support^2^. Only the Statens Serum Institut in Denmark continued to produce the TST when the production of tuberculin in Brazil was completely stopped. Since then, the Brazilian public health system (SUS) has experienced regular shortages of this skin test, also known as PPD^3^.

ANVISA’s new biosafety regulations require TST to be produced in a biosafety level 3 (BSL-3) laboratory, but the production of LST requires only a BSL-2 structure. As a result, a lower investment over a shorter period was required to adapt the CPPI manufacturing plant (personal communication, Fabiano Figueiredo, ICC/Fiocruz). However, the CPPI, a public laboratory in the state of Paraná, does not have the necessary capital (approximately 30 million BRL) to produce GMP-grade leishmanin. A severe economic crisis in 2015, combined with the inherent lack of political interest in the category of neglected tropical diseases (NTDs), which includes leishmaniasis, finally halted production of the Montenegro test at the CPPI in 2016.

The only social group that could demand that leishmaniasis be put back on the political agenda for science and technology are experts, clinicians, and other investigators who work with this disease daily. These leishmaniacs, as they call themselves, participate in various meetings and networks related to decision making and priority setting, including ChagasLeish, the Brazilian Society for Tropical Medicine, and redeLEISH. It is somewhat surprising that this group did not advocate the resumption of LST production, either in redeLEISH or in the 2016 closed meetings organized by the Brazilian Ministry of Health and PAHO, which I had the honor of attending. Instead, they highlighted the need for a new diagnostic test for cutaneous leishmaniasis.

The technological and scientific advances in this field are so obvious that scholars call the current age the fourth industrial revolution or the age of digital healthcare^4^. Various diagnostic tests can be performed today in pharmacies or at home. However, if we can continuously monitor biological parameters through our cell phones, why cannot we imagine the same technological progress for leishmaniasis? Leishmaniasis experts seem to believe that LST is no longer needed because new generations of tests will diagnose cutaneous leishmaniasis more accurately.

Our society is described as future-oriented because it tends to reject anything that does not reach a certain level of technological performance^5^. While we are collectively convinced that technological progress is the only path to follow, we forget that this progress is conditioned by political and economic arrangements. Technology cannot be considered separately from the social and economic context of NTDs. Frankly, this classification denotes diseases that do not create a market (without incentives); in other words, their market potential is insufficient to readily attract private sector investment. Therefore, longer periods are required for the introduction of new technologies in this market than for other categories of diseases. Moreover, some innovations will never reach patients simply because no industrial partners are willing to manufacture them. Generally, no new product can emerge unless there is a profitable market and/or strong institutions to ensure that the investment in the new technology is worthwhile. National governments must be involved in every phase of development and planning of scientific and technological innovations related to NTDs to ensure a market (in the case of Brazil: SUS). 

Future-oriented discourses and blind faith in modernity distract us from the possibility of advocating a simple product, the Montenegro skin test. We can strive for better diagnostic tools, but in the meantime, the Brazilian government must protect LST. Brazil has the infrastructure and capacity in some public laboratories to produce GMP-grade quality leishmanin. Bio-Manguinhos, the Institute of Technology on Immunobiologicals, is one example; in fact, this public laboratory was the second-largest supplier of the Montenegro skin test to the Brazilian Ministry of Health until 2003, when the CPPI became the only supplier. Bio-Manguinhos stopped producing the LST because it is so inexpensive and simple to manufacture, preferring to focus on higher value-added products that augment revenue and streamline the manufacturing process^6^. The LST is indeed very cheap: in 2015, the Ministry of Health paid only 7.00 BRL for each kit^7^.

This may explain why LST is no longer manufactured worldwide, but this test can still make a difference in many social and/or remote settings because it is inexpensive and easy to use^8^. With a relatively small investment, Brazil could become the world's only producer of GMP-grade leishmanin and export this product to other endemic countries such as Iran, Morocco, and Tunisia. According to Romero^9^, LST is a strategic health product. Now we must convince the "leishmaniacs" that high-tech products are not always the solution. As the history of tropical diseases shows, simple and inexpensive solutions have often saved Brazil.
